# The rs243865 Polymorphism in Matrix Metalloproteinase-2 and its Association With Target Organ Damage in Patients With Resistant Hypertension: Cross-Sectional Study

**DOI:** 10.2196/71016

**Published:** 2025-05-01

**Authors:** An Tuan Huynh, Hoang Anh Vu, Ho Quoc Chuong, Tien Hoang Anh, An Viet Tran

**Affiliations:** 1 Can Tho University of Medicine and Pharmacy, 179 Nguyen Van Cu, An Khanh, Ninh Kieu, Can Tho, Vietnam, 84 939060818; 2 Center for Molecular Biomedicine, University of Medicine and Pharmacy at Ho Chi Minh City, Ho Chi Minh City, Vietnam; 3 Huế University of Medicine and Pharmacy, Huế, Vietnam

**Keywords:** resistant hypertension, matrix metalloproteinase-2, gene polymorphism, target organ damage, arterial stiffness

## Abstract

**Background:**

Resistant hypertension (RH) presents significant clinical challenges, often precipitating a spectrum of cardiovascular complications. Particular attention recently has focused on the role of matrix metalloproteinase-2 (MMP-2) gene polymorphisms, implicated in hypertensive target organ damage (TOD). Despite growing interest, the specific contribution of MMP-2 polymorphisms to such damage in RH remains inadequately defined.

**Objective:**

This study is the first to examine the rs243865 (−1306C>T) polymorphism in the MMP-2 gene in the Vietnamese population and patients with RH, underscoring its critical role as a genetic determinant of TOD.

**Methods:**

A cross-sectional study with both descriptive and analytical components was conducted with 78 patients with RH at the Can Tho Central General Hospital and Can Tho University of Medicine and Pharmacy Hospital from July 2023 to February 2024.

**Results:**

More than three-quarters of patients with RH had carotid-femoral pulse wave velocity (PWV) >10 m/s and microalbuminuria at a prevalence of 79% (62/78) and 76% (59/78), respectively, and more than half of patients with RH had left ventricular mass index, relative wall thickness, and carotid artery stenosis with a prevalence of 56% (45/78), 55% (43/78), and 53% (41/78), respectively. Of the 78 studied patients with RH, the presence of genotype CC was 77% (60/78), genotype CT accounted for 21% (16/78), and genotype TT for 3% (2/78). The presence of single nucleotide polymorphism rs243865 (−1306C>T) with allele T was 23% (18/78). The MMP-2 gene polymorphism 1306C/T (rs243865) was significantly associated with ejection fraction and carotid artery stenosis with odds ratios (ORs) 8.1 (95% CI 1.3‐51.4; *P*=.03) and 4.5 (95% CI 1.1‐20.1; *P*=.048), respectively. The allele T was found to be significantly associated with arterial stiffness including brachial-ankle PWV and carotid-femoral PWV with the correlation coefficient of OR 2.2 (95% CI 0.6‐3.8) and OR 1.8 (95% CI 0.5‐3.2), respectively.

**Conclusions:**

The MMP-2 gene polymorphism rs243865 (−1306C>T) may have an association with measurable TOD in RH.

## Introduction

Resistant hypertension (RH) is characterized by the inability to achieve optimal blood pressure (BP) control despite the administration of maximum tolerated doses of antihypertensive medications, including a diuretic. This condition presents a significant clinical challenge, as it is influenced by a multitude of genetic, environmental, and pathophysiological factors that contribute to persistent hypertension. RH is closely associated with severe target organ damage (TOD), which includes damage to the heart, kidneys, and vasculature, significantly increasing the risk of cardiovascular events and mortality. Despite advancements in antihypertensive therapies, approximately 70% of patients with hypertension fail to achieve recommended BP targets, underscoring the complexity of this condition [1].

Among the molecular mechanisms contributing to RH, matrix metalloproteinases (MMPs), particularly the gelatinase family (MMP-2, MMP-9), have garnered considerable attention. These enzymes play a critical role in extracellular matrix (ECM) remodeling, a process essential to the pathogenesis of several cardiovascular diseases such as coronary artery disease, arteriosclerosis, and systemic hypertension [2]. MMP-2, in particular, has been implicated in the remodeling of cardiovascular tissues, contributing to vascular stiffness and fibrosis, both of which are key contributors to RH and TOD [3]. Recent studies have focused on the genetic variants of the MMP-2 gene, especially single nucleotide polymorphisms (SNPs), and their potential role in the development and progression of cardiovascular diseases [4-6]. These genetic polymorphisms are believed to modulate MMP-2 expression and activity, thereby influencing the extent of cardiovascular remodeling and associated TOD. Given the growing evidence linking MMP-2 activity with hypertension-related TOD, understanding the genetic underpinnings of MMP-2 in RH could offer new insights into disease mechanisms and therapeutic targets. The objectives of this study are: (1) to investigate the clinical characteristics and extent of TOD in patients with RH; and (2) to determine the polymorphisms of the MMP-2 gene and assess their association with TOD in patients with RH.

## Methods

### Study Population

This study focused on patients with hypertension admitted to Can Tho Central General Hospital and Can Tho University of Medicine and Pharmacy Hospital from July 2023 to February 2024. The study population was divided into 2 groups: patients with RH and patients with well-controlled hypertension. The diagnosis of RH followed the 2021 guidelines of the Vietnam Hypertension Society [7].

### Sample Size

#### Overview

To achieve the objective: “Determining the polymorphism of rs243865 and its association with TOD in patients with RH,” we used the formula for estimating a single proportion. The sample size was estimated using the following formula:


$$n=Z_{1-a/2}^{2}\frac{p(1-p)}{d^{2}}$$

where n=required sample size; Z=*z* score corresponding to a 95% CI (*z*=1.96); d=desired margin of error (chosen as d=0.1); and p=proportion of patients carrying the minor allele T in the RH group, estimated at 25%.

Applying the values to the formula yielded a required sample size of 72 patients with RH. In practice, 78 patients were enrolled.

#### Inclusion Criteria

Adults aged 18 years or older diagnosed with RH, defined as the failure to achieve target BP (systolic <140 mm Hg or diastolic <90 mm Hg) despite the use of optimal or best-tolerated doses of 3 or more antihypertensive medications, including a diuretic, with BP inadequately controlled as confirmed through home or ambulatory BP monitoring, and without secondary causes of hypertension or evidence of pseudoresistant hypertension.

#### Exclusion Criteria

Patients were excluded from the study if they had any of the following conditions: acute medical emergencies, active autoimmune diseases or ongoing immunosuppressive therapies, cancer or other malignant conditions, secondary hypertension confirmed by clinical and laboratory examinations, pregnancy or chronic kidney disease (CKD), or if they refused to participate or demonstrated nonadherence to the medication regimen.

### Methodological Approach

#### Design Framework

The study used a cross-sectional, descriptive-analytic design to investigate the association between the SNP rs243865 (−1306C>T) in the MMP-2 gene and RH versus nonresistant hypertension. Patients were recruited from 2 hospitals from July 2023 to February 2024. Patients were classified into resistant and nonresistant hypertension groups according to the European Society of Cardiology criteria for RH.

#### Sampling Strategy

Nonprobability convenience sampling method was used. Patients meeting inclusion criteria were recruited consecutively upon admission to the cardiology and internal medicine departments. Trained research assistants approached patients daily, explained the study objectives, and obtained informed consent prior to enrollment. Convenience sampling was selected due to logistical feasibility and time constraints.

### Research Protocol and Variables

#### Demographic and Risk Factors

Data were systematically collected regarding the following risk factors and comorbid conditions, clearly defined based on standard clinical criteria:

- Diabetes mellitus: defined as having a documented diagnosis of diabetes, or current use of antidiabetic medications, or fasting plasma glucose ≥126 mg/dL, or HbA_1c_≥6.5%.
- Overweight or obesity: defined according to BMI classification, with overweight as BMI≥25 kg/m² and obesity as BMI≥30 kg/m², calculated from measured height and weight.
- Smoking status: categorized as smoker (currently smoking ≥1 cigarette per day or having ceased smoking for at least 6 mo prior to enrollment), or nonsmoker (no lifetime smoking).
- History of heavy drinking: defined according to the National Institute on Alcohol Abuse and Alcoholism guidelines as consumption of ≥14 drinks per week for men or ≥7 drinks per week for women, or a documented history of alcohol use disorder.

These data were obtained through structured patient interviews and cross-verified by medical records to ensure accuracy and consistency.

#### Clinical and Hemodynamic Parameters

##### Overview

BP and pulse pressure were measured using the BOSO ABI-100 system in all patients to minimize errors, with measurements taken at least twice in a seated position after 5 minutes of rest; pulse pressure was calculated as the difference between systolic and diastolic BP [8]. A 24-hour ambulatory BP monitoring device was used to assess mean systolic and diastolic BP, nocturnal dipping, and early morning BP surge. The resting heart rate was measured manually or with a digital monitor. Blood samples were collected to determine serum levels of urea, creatinine, and electrolytes, including sodium, potassium, and chloride. TOD was evaluated across several key organs, with specific diagnostic criteria used to define damage in each organ system.

##### Cardiac Damage

Left ventricular hypertrophy (LVH) was assessed using echocardiography, with the left ventricular mass index (LVMI) calculated. According to the European Society of Cardiology guidelines, LVH was defined as LVMI >95 g/m² for women and LVMI >115 g/m² for men. Electrocardiogram criteria for LVH, such as the Sokolow-Lyon and Cornell voltage criteria, were also used as secondary diagnostic tools [1].

Left ventricular ejection fraction (EF), a key indicator of cardiac function, was measured via echocardiography. EF was classified as normal (≥50%), mildly reduced (41%‐49%), moderately reduced (30%‐40%), or severely reduced (<30%). All the echocardiography is made via Siemens Acuson X300 ultrasound machine.

##### Brain Damage

Brain damage was assessed through imaging techniques, including computed tomography and magnetic resonance imaging. The presence of any of the following conditions was considered indicative of brain damage: white matter lesions, cerebral microbleeds, lacunar infarctions, and dilated perivascular spaces.

A history of stroke or transient ischemic attack was also considered as evidence of brain damage.

##### Renal Damage

Renal damage was assessed using the urinary albumin-to-creatinine ratio. This method evaluates kidney function by measuring albumin excretion in the urine.

Renal damage was defined as an albumin-to-creatinine ratio of: normal to mildly increased (<30 mg/g); moderately increased (30‐300 mg/g); and severely increased (>300 mg/g).

Patients with a history of CKD stage 4 or 5, or renal failure (estimated glomerular filtration rate<30 mL/min/1.73 m²), were excluded from the study to avoid confounding factors related to advanced renal failure.

##### Vascular Damage

Vascular stiffness was assessed using pulse wave velocity (PWV), defined as the speed at which arterial pressure waves move along the vessel wall, with a PWV >10 m/s being indicative of vascular damage via the BOSO ABI-100 system. The ankle-brachial index (ABI) was also measured using the BOSO ABI-100 system. ABI is defined as the ratio of the systolic BP measured at the ankle to the systolic BP measured at the brachial artery. An ABI of ≤0.9 was indicative of peripheral arterial disease and thus considered a sign of vascular damage.

Carotid artery damage was assessed using ultrasound to measure carotid intima-media thickness. Carotid stenosis was defined as the presence of plaques that caused a ≥50% reduction in the arterial lumen or if the intima-media thickness was ≥0.9 mm. Significant stenosis was confirmed through Doppler ultrasound via Siemens Acuson X300 ultrasound machine.

### MMP-2 Gene Polymorphism Analysis

#### Sequencing Technique

A 4 mL blood sample was collected into ethylenediaminetetraacetic acid–coated tubes and stored at 2 °C until used for DNA extraction and analysis. The SNP genotype was determined using 2 direct sequencing methods.

#### Principle

The sequencing technique was carried out using an automated sequencer based on a modified Sanger method. In this method, the dideoxynucleotide triphosphates are not radioactively labeled but are tagged with different fluorescent dyes for each type of dideoxynucleotide triphosphate. The automated sequencer comprises key components such as a capillary system, a laser illumination system, and a signal detection and processing system. The capillary electrophoresis bands emit light as they pass through a laser beam, and the color detection system records and encodes the nucleotides as A, T, C, or G.

#### Main Steps in Sequencing

DNA extraction was performed using the Qiagen DNA extraction kit (Qiagen, Hilden, Germany), following the manufacturer’s protocol. The target region containing the SNP was then amplified via polymerase chain reaction (PCR). The PCR products were visualized through agarose gel electrophoresis, and subsequently purified using the Qiagen PCR purification kit (Qiagen, Hilden, Germany). Sequencing of the purified PCR products was carried out using the modified Sanger method. Capillary electrophoresis was performed on a Beckman Coulter CEQ8000 sequencer. The sequence data were further analyzed using the ABI 3500 Genetic Analyzer (Applied Biosystems, Foster City, California, United States). Sequence processing and SNP analysis were conducted with SeqScape software (version 2.7; Applied Biosystems). The results were interpreted by comparing the identified SNP locations with the corresponding reference sequences retrieved from the National Center for Biotechnology Information database.

#### Method

Sequencing was performed using the Beckman Coulter CEQ8000 sequencer.

### Statistical Analysis

The dataset underwent statistical treatment using Stata (version 15.1; StataCorp) and was articulated through frequency distribution (for qualitative variables), and mean (SD; for quantitative measures). Comparison for qualitative data was made by chi-square tests and by 2-tailed Student *t* tests for quantitative data. A signiﬁcance level of .05 was used for all tests to establish statistical signiﬁcance. Stepwise multiple regression analysis with inclusion at the .01 level was used to evaluate the influence of gen rs243865 (−1306C>T) on targeted organ damage adjusted by clinical and subclinical characteristics. To estimate the relationship between MMP-2 gene SNPs and TOD, odds ratio and its 95% CI were calculated for binary TOD variables including echocardiogram EF and carotid artery stenosis. Regression coefficients (β reg coef.) and its 95% CI were calculated for continuous TOD variables including brachial-ankle PWV (m/s) and carotid-femoral PWV (cfPWV; m/s). The squared correlation coefficient (*R*^2^) was calculated for the proportion of variance explained by the model.

### Ethical Considerations

The study was approved by the Ethics Council in Biomedical Research, Can Tho University of Medicine and Pharmacy, through the research ethics approval form 23.006.NCS/HĐĐĐ dated June 15, 2023, before data collection. The study was also licensed to be conducted at Can Tho Central General Hospital and Can Tho University of Medicine and Pharmacy Hospital. The study was conducted with the consent of the participants through the consent form. The process of interview and the implementation of testing techniques were conducted conveniently and comfortably for the participants, not related to private issues that may affect the health or psychology of the participants. Participants did not receive any compensation for their participation. The personal information of the participants was kept confidential. This study aimed to protect and improve public health and has no other purpose.

## Results

**Figure 1.**
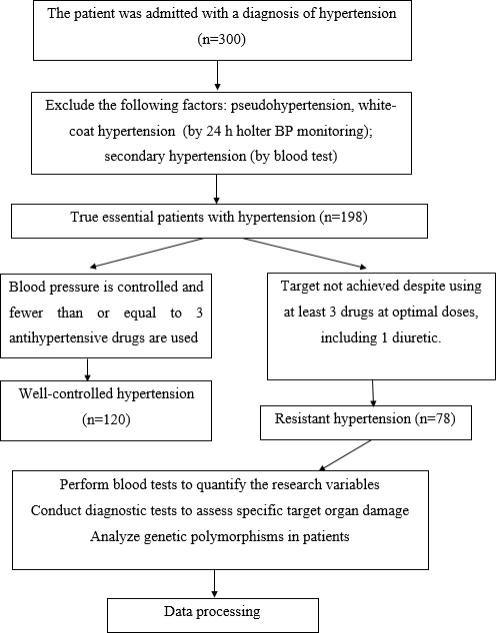
Study protocol. BP: blood pressure.

The protocol is presented in the study diagram (Figure 1). In our analysis of 78 patients with RH, a significant proportion were female (49/78, 63%), with an average age of 66.7 (SD 14.4) years. The majority of patients (51/78, 65%) were older than 60 years of age, highlighting the predominance of an older cohort. Notably, 68% (53/78) of the patients had a history of hypertension extending beyond 10 years, reflecting the chronic nature of RH, which complicates BP control (Table 1).

**Table 1.** Clinical characteristics of patients with RH^a^.

Clinical characteristics	Value (N=78), n (%)
Sex	
Male	29 (37)
Female	49 (63)
Age^b^ (years)	
≤60	27 (35)
>60	51 (65)
Duration of hypertension^c^ (years)	
≤10	53 (68)
>10	25 (32)
Blood pressure level	
Grades 1 and 2	53 (68)
Grade 3	25 (32)
Diabetes	
Yes	22 (28)
No	56 (72)
Overweight or obese	
Yes	20 (26)
No	58 (74)
Smoking (current or past history)	
Yes	24 (31)
No	54 (69)
History of heavy drinking	
Yes	25 (32)
No	53 (68)
Triglyceride^d^ (mmol/L)	
≥2.26	38 (49)
<2.26	40 (51)
LDL^e^ (mmol/L)^f^	
≥3.36	24 (31)
<3.36	54 (69)
Blood lipid disorders	
Yes	49 (63)
No	29 (37)

a RH: resistant hypertension.

b Age: mean 66.7 (SD 14.4) years.

c Duration of hypertension: mean 10.3 (SD 5.6).

d Triglyceride: mean 2.85 (SD 2.42)

e LDL: low-density lipoprotein.

f LDL: mean 2.95 (SD 1.28)

Despite treatment adherence, mean systolic and diastolic BP levels were persistently elevated, averaging 162.5 (SD 29.6) mm Hg and 92.7 (SD 15.9) mm Hg, respectively. This underscores the therapeutic challenges posed by RH. Common comorbidities included diabetes (22/78, 28%) and obesity (20/78, 26%). Additionally, dyslipidemia was prevalent, with high serum triglycerides (38/78, 49%) and low-density lipoprotein cholesterol (24/78, 31%). The prevalence of TOD was striking, with 79% (62/78) of patients demonstrating cfPWV >10 m/s, an indicator of increased arterial stiffness. Microalbuminuria, found in 76% (59/78) of patients, suggests significant renal impairment, while over half of the cohort showed elevated LVMI and increased relative wall thickness, both markers of adverse cardiac remodeling driven by chronic hypertension (Table S1 in Multimedia Appendix 1).

The MMP-2 gene polymorphism rs243865 (−1306C>T) was investigated, revealing that 77% (60/78) of patients carried the CC genotype, while 21% (16/78) carried the CT genotype, and 3% (2/78) the TT genotype (Table 2). The T allele frequency was 23% (18/78), potentially highlighting a genetic predisposition for more severe vascular outcomes in RH.

**Table 2.** Distribution of MMP-2^a^ gene polymorphism rs243865 (−1306C>T) in patients with RH^b^.

MMP-2 gene polymorphism rs243865 (−1306C>T)		Value (N=78), n (%)
Genotype		
CC		60 (77)
CT		16 (21)
TT		2 (3)
Allele		
T carrier		18 (23)
CC		60 (77)

a MMP-2: matrix metalloproteinase-2.

b RH: resistant hypertension.

Significant relationships were identified between the T allele and specific TOD markers, particularly reduced EF and increased cfPWV. T allele carriers exhibited a lower mean EF (53.8, SD 20.3) compared to noncarriers (62.1, SD 12.7), with a statistically significant difference (*P*=.04). Additionally, T allele carriers had higher brachial-ankle PWV and cfPWV values, nearing statistical significance (both *P*=.07), suggestive of enhanced arterial stiffness (Table 3).

**Table 3.** The comparison mean of target organ damage indicators between MMP-2^a^–carrying polymorphisms nucleotide at rs243865 (-1306C>T) with and without allele T.

Indicators of target organ damage	T carrier (n=18), mean (SD)	CC (n=60), mean (SD)	*P* value^b^
Left ventricular mass index (g/m^2^)	120.1 (55.9)	114.9 (44.9)	.69
EF^c^ in echocardiogram	53.8 (20.3)	62.1 (12.7)	.04
Blood pressure difference	70.3 (15.5)	71.4 (22.1)	.84
ABI^d^	0.98 (0.15)	0.99 (0.2)	.76
Brachial-ankle PWV^e^ (m/s)	19.1 (3.5)	17.4 (3.5)	.07
Carotid-femoral PWV (m/s)	13.6 (2.9)	12.2 (2.9)	.07
eGFR^f^	66.6 (27.2)	74.4 (32.3)	.36
ACR^g^	130.2 (147.7)	140.5 (182.9)	.84

a MMP-2: matrix metalloproteinase-2.

b *P* value: independent samples 2-tailed *t* test.

c EF: ejection fraction.

d ABI: ankle-brachial index.

e PWV: pulse wave velocity.

f eGFR: estimated glomerular filtration rate.

g ACR: albumin-to-creatinine ratio.

The association between the T allele and carotid artery stenosis was also notable, with 72% (13/18) of T allele carriers exhibiting stenosis compared to 47% (28/60) of noncarriers, approaching statistical significance (*P*=.06; Table 4). T allele carriers exhibited a higher prevalence of EF of <40% and carotid artery stenosis compared to noncarriers (Table 5). Specifically, 22% (4/18) of T allele carriers had an EF of <40%, compared to only 7% (4/60) of noncarriers, approaching statistical significance (*P*=.06). Similarly, carotid artery stenosis was present in 72% (13/18) of T allele carriers versus 47% (28/60) of noncarriers (*P*=.06), indicating a potential role of the T allele in exacerbating arterial remodeling and stenosis (Table 4). After adjusting for age and serum potassium levels, the T allele remained significantly associated with EF <40% (Table 5). After adjusting for age, hypertension duration, and sodium levels, T allele carriers had a significantly higher risk of carotid artery stenosis (Table S2 in Multimedia Appendix 1).

**Table 4.** The comparison of the percentage of hypertension-mediate organ damage between MMP-2^a^ polymorphisms nucleotide at rs243865 (−1306C>T) with and without allele T.

Symptoms of target organ damage	T carrier (n=18), n (%)	CC (n=60), n (%)	*P* value^b^
History of stroke or TIA^c^	4 (22)	14 (23)	.92
ECG^d^ ischemia	9 (50)	18 (30)	.12
ECG left ventricular hypertrophy	4 (22)	13 (22)	.96
Echocardiogram EF^e^ <40%	4 (22)	4 (7)	.06
Echocardiogram with regional hypokinesis	6 (33)	22 (38)	.79
Echocardiographic left ventricular mass index (>95 for women and >115 for men)	9 (50)	35 (58)	.53
Echocardiographic relative wall thickness ≥0.43	10 (56)	33 (55)	.97
Carotid artery stenosis	13 (72)	28 (47)	.06
Ankle-brachial index <0.9	3 (17)	11 (18)	.87
Carotid-femoral pulse wave velocity >10 m/s	16 (89)	45 (75)	.21
eGFR^f^ <60 mL/min/1.73m^2^	7 (39)	17 (28)	.39
Albuminuria (urine albumin/creatinine ratio >30 µg/g)	14 (78)	45 (75)	.81

a MMP-2: matrix metalloproteinase-2.

b *P* value: chi-square.

c TIA: transient ischemic attack.

d ECG: electrocardiogram.

e EF: ejection fraction.

f eGFR: estimated glomerular filtration rate.

**Table 5.** Association of MMP-2^a^ gene polymorphism rs243865 (−1306C>T) and echocardiogram EF^b^ in resistant hypertension (N=78).

		EF <40%	EF ≥40%	Univariate logistic regression		Multivariate logistic regression^c^	
				OR^d^ (95% CI)	*P* value	OR (95% CI)	*P* value
rs243865 (−1306C>T), n (%)					.06		.03
T Carrier		4 (22)	14 (78)	4.0 (0.9-18.0)		8.1 (1.3‐51.4)	
CC		4 (7)	56 (93)	—^e^		—	
Age group (years), n (%)					.09		.06
≤60		5 (19)	22 (82)	—		—	—
≥61		3 (6)	48 (94)	0.3 (0.06‐1.3)		0.2 (0.03‐1.1)	
Potassium serum concentration, mean (SD)		3.3 (0.3)	3.6 (0.4)	0.13 (0.14‐1.2)	.06	0.1 (0.01‐1.3)	.07

a MMP-2: matrix metalloproteinase-2.

b EF: ejection fraction.

c The 3-factor model *R*2=0.2306.

d OR: odds ratio.

e Not applicable.

The T allele was also associated with higher cfPWV, a marker of arterial stiffness and a predictor of cardiovascular events (Table S3 in Multimedia Appendix 1). The multivariate regression model showed a significant correlation between the T allele and increased PWV (β=1.8, 95% CI 0.5‐3.2; *P*=.008). This highlights the potential role of the rs243865 polymorphism in promoting arterial stiffness.

## Discussion

### Principal Findings

In this study, we selected patients with true RH, excluding those with advanced-stage CKD and secondary hypertension. This ensured that the TOD observed was specific to patients with primary hypertension, a population that typically receives inadequate screening for TOD. Our patient cohort, representing the health care setting of a resource-limited country, included a predominantly lower-income population. These patients often exhibit limited concern for their health and lack access to regular check-ups compared to those in high-income countries. Our findings, which were largely anticipated, emphasize several critical characteristics and clinical implications of RH. These include the difficulty in controlling BP, its association with comorbidities, and the significant burden of TOD, consistent with prior studies over the past 5 years.

### Comparison to Prior Work

#### Demographic and Clinical Characteristics

The predominance of female patients (49/78, 63%) and older patients (51/78, 65% older than 60 years of age) is consistent with previous research showing that RH is more prevalent among older adults and female patients [9,10]. A history of hypertension exceeding 10 years in 68% (53/78) of patients reflects the chronic nature of the condition, which not only complicates BP management but also elevates the risk of TOD [11].

Despite adherence to treatment, mean systolic and diastolic BP levels remained high (162.5, SD 29.6 mm Hg and 92.7, SD 15.9 mm Hg, respectively). This highlights the challenges of achieving BP targets in RH, which may be attributed to inflammatory mechanisms and hyperactivity of the sympathetic nervous system and the renin-angiotensin-aldosterone system [1].

The high prevalence of diabetes (22/78, 28%) and obesity (20/78, 26%) in this cohort aligns with well-established risk factors for RH. These conditions not only contribute to endothelial dysfunction but also exacerbate arterial stiffness, worsening hypertension [12,13]. Dyslipidemia, characterized by elevated triglycerides (38/78, 49%) and low-density lipoprotein cholesterol (24/78, 31%), further increases cardiovascular risk and TOD [14]. Although diabetes and obesity are not considered primary causes of secondary hypertension, effective management of weight and glucose levels can improve BP control and overall prognosis in patients with RH.

#### TOD

The burden of TOD in patients with RH was substantial. A high proportion of patients 79% (62/78) demonstrated elevated cfPWV (>10 m/s), indicating significant arterial stiffness—a critical marker of vascular aging and cardiovascular risk [15]. While cfPWV is predominantly used in research settings rather than routine clinical practice, it remains a robust prognostic indicator independent of brachial BP. Interestingly, we observed that cfPWV does not always correlate with BP levels, suggesting that relying solely on BP measurements may overlook high-risk patients with significant arterial stiffness. The high prevalence of elevated cfPWV in this study could be both a consequence of prolonged hypertension and a contributing factor to RH.

Microalbuminuria was observed in 76% (59/78) of patients, indicating early renal dysfunction and its central role in RH pathophysiology via sodium retention and renin-angiotensin-aldosterone system activation [11,16]. While most clinicians rely on creatinine levels and estimated glomerular filtration rate to assess renal damage, our findings reveal a concerning rate of early kidney damage even in patients without advanced CKD, warranting greater clinical attention.

LVH and increased relative wall thickness were observed in over half of the patients, consistent with previous studies highlighting the importance of echocardiography in accurately assessing cardiac TOD. Compared to electrocardiograms, echocardiography has significantly higher sensitivity in detecting LVH [16-18].

Furthermore, RH has been shown to substantially increase the risk of severe cardiovascular events, including heart failure, myocardial infarction, and stroke, particularly in ambulatory RH cases [14].

#### Association of SNP With TOD

Our analysis demonstrates a strong association between the rs243865 (−1306C>T) polymorphism in the MMP-2 gene and TOD in patients with RH. The results emphasize that the T allele (the minor allele) significantly increases the risk of arterial stiffness, carotid artery stenosis, and reduced EF. Previous studies have shown that rs243865 enhances the transcriptional activity of MMP-2, leading to excessive ECM degradation, which contributes to vascular and cardiac fibrosis [19,20].

In this study, cfPWV, a key indicator of arterial stiffness, was on average 1.8 m/s higher in the T allele group compared to the CC genotype group. This aligns with previous finding [21], which highlighted the critical role of MMP-2 in promoting arterial fibrosis, particularly in older individuals. Other studies also indicated that MMP-2 polymorphisms are associated with increased arterial stiffness in hypertensive populations [22,23]. Furthermore, inflammation and oxidative stress interact with MMP-2 activity, exacerbating arterial stiffness in patients with RH [24]. Evidence from multiple studies indicates that arterial stiffness is independently linked to genetic factors, irrespective of BP control, paving the way for its potential as a predictive marker for resistance to antihypertensive therapy [3,21,24].

The prevalence of carotid artery stenosis was significantly higher in the T allele group, underscoring its critical role in vascular remodeling. Our findings are consistent with previous studies, which demonstrated that rs243865 upregulates MMP-2, promoting the development of atherosclerotic plaques and narrowing the arterial lumen [19,25]. Additionally, ECM remodeling mediated by MMP-2 reduces arterial elasticity and contributes to carotid artery stenosis [26]. However, prior studies emphasized that beyond rs243865, other genetic and environmental factors play a critical role, reflecting the multifactorial nature of this pathology [27].

Patients carrying the T allele exhibited significantly lower EF, with an average reduction of approximately 8% compared to the CC genotype group, indicating impaired cardiac function and an increased risk of heart failure. Previous studies have reported that haplotypes in the MMP-2 gene are associated with LVH, myocardial infarction, and impaired cardiac function [28,29]. The enhanced activity of MMP-2 driven by rs243865 leads to ECM degradation, destabilizing cardiac structure and triggering compensatory fibrosis. This finding presents a potential therapeutic application, as the inhibition of MMP-2 has been shown to improve cardiac function in preclinical models [30]. From a broader perspective on causality, reduced EF often originates from pressure overload and vascular remodeling. The influence of the MMP-2 gene on vascular structure, leading to arterial stiffness, may impair cardiac function by increasing afterload [21].

#### The Role of Genetics in TOD

This study, aligned with previous studies, highlights the significant role of the rs243865 (−1306C>T) polymorphism in the MMP-2 gene in the risk of TOD [31]. This genetic variant not only exerts its effects independently but also interacts intricately with other factors such as inflammation and environmental influences. Specifically, this polymorphism increases the risks of arterial stiffness, carotid artery stenosis, and impaired cardiac function in patients with RH. Genetic variants within the MMP-2 gene can significantly alter the risk of cardiovascular diseases [5,23]. These variants play a pivotal role in vascular remodeling, leading to severe outcomes such as LVH and reduced cardiac pumping capacity. The rs243865 polymorphism, through enhanced MMP-2 activity, disrupts ECM integrity, thereby contributing to the structural weakening of the vasculature and heart [32]. Furthermore, rs243865 has been implicated in other vascular diseases beyond hypertension, including ischemic stroke and aneurysms. This underscores its potential as a critical risk factor in systemic vascular conditions. The overactivation of MMP-2 associated with rs243865 leads to excessive ECM degradation, weakening vascular structures and promoting the development and progression of vascular lesions [4,33]. Recently, intermediate factors, such as obesity and insufficient physical activity, proved capable of amplifying the effects of rs243865 on BP and TOD [6]. Obesity, through mechanisms of chronic inflammation and endocrine disruption, exacerbates MMP-2 activity, while sedentary lifestyles further contribute to vascular dysfunction [27]. Synthesizing all these findings, rs243865 emerges as not only a key genetic determinant of TOD but also a nexus of complex interactions with other factors, including inflammation, oxidative stress, lifestyle, and environmental influences. This highlights its potential as a target for personalized treatment strategies aimed at regulating MMP-2 activity and mitigating its associated impacts in the management of RH.

### Limitations

This study is limited by its small sample size, cross-sectional design, and focus on a single ethnic population, which may affect the generalizability of the findings. Additionally, unmeasured confounding factors, such as inflammation and interactions with other genetic polymorphisms, were not assessed. Further longitudinal and multiethnic studies are needed to validate these results and explore the broader implications of rs243865 and TOD in RH. First, this study used a relatively small sample size (N=78), which may limit the generalizability and statistical power of our findings. To mitigate this, we calculated the sample size based on a statistically valid estimation formula to ensure adequate representation; however, larger multicenter studies would enhance statistical power. Second, the cross-sectional design of this study prevents us from establishing a causal relationship between the rs243865 polymorphism and TOD. While this design enabled the identification of associations, longitudinal studies would be necessary to clarify causality and the temporal sequence of events. Third, although this is the first study about rs243865 in Vietnamese people, the focus on a single ethnic group limits the external validity of the findings, potentially restricting applicability to other populations. To address this, future research should include diverse ethnic groups to assess whether these genetic associations hold across different populations. Finally, due to limited data availability, we were unable to compare the genotype distribution of rs243865 in our patients with RH with that in the general Vietnamese population. This limitation should be addressed in future population-based studies to provide a more comprehensive interpretation of the genetic findings.

### Future Directions

Future research could expand the scope by exploring additional genetic polymorphisms within the MMP-2 gene and their combined impact with rs243865 on RH and associated TOD. Translating findings from genetic associations into clinical practice represents a significant opportunity. Genetic screening for MMP-2 polymorphisms could facilitate personalized medicine approaches by identifying patients at higher risk for RH and severe TOD, allowing clinicians to initiate more aggressive or targeted interventions earlier in the treatment course. Additionally, therapeutic strategies targeting MMP-2 activity, such as the use of specific inhibitors, may offer new avenues for managing and mitigating vascular and cardiac complications in patients with RH and patients with cardiovascular disease as in our prior study [34].

### Conclusions

This study underscores the critical role of the rs243865 (−1306C>T) polymorphism in the MMP-2 gene as a significant genetic determinant of TOD in patients with RH. Our findings highlight the multifaceted impact of this polymorphism, including its association with increased arterial stiffness, carotid artery stenosis, and reduced EF. Importantly, the influence of rs243865 extends beyond its direct genetic effects, interacting with inflammation, oxidative stress, and modifiable factors such as obesity and physical activity. The high prevalence of TOD in our patient population underscores the urgent need for comprehensive screening and management strategies, particularly in resource-limited settings where access to advanced diagnostic tools remains a challenge.

The study provides compelling evidence for considering rs243865 as a potential biomarker for risk stratification and a target for therapeutic intervention. Future research should focus on validating these findings in larger and more diverse populations, exploring the mechanistic pathways linking MMP-2 activity to TOD, and evaluating the clinical efficacy of MMP-2 inhibitors in reducing vascular and cardiac complications in patients with RH. Moreover, integrating genetic testing for rs243865 into clinical practice could pave the way for personalized treatment approaches, allowing for more targeted and effective management strategies.

## Supplementary material

10.2196/71016 Multimedia Appendix 1 Supplementary tables.

## Abbreviations

ABI: ankle-brachial index
BP: blood pressure
cfPWV: carotid-femoral pulse wave velocity
CKD: chronic kidney disease
ECM: extracellular matrix
EF: ejection fraction
LVH: left ventricular hypertrophy
LVMI: left ventricular mass index
MMP: matrix metalloproteinase
PCR: polymerase chain reaction
PWV: pulse wave velocity
RH: resistant hypertension
SNP: single nucleotide polymorphism
TOD: target organ damage
