# Supplementary material for: The rs243865 Polymorphism in Matrix Metalloproteinase-2 and its Association With Target Organ Damage in Patients With Resistant Hypertension: Cross-Sectional Study
Source: JMIR Cardio. 2025 May 1;9:e71016. doi: 10.2196/71016 (PMC12061201; doi:10.2196/71016)
Supplement: Multimedia Appendix 1 [file cardio-v9-e71016-s001.docx]

**Table S1.** Characteristics of TOD indicators of patients with RH.

| **Characteristics (n=78)** | **Mean±SD** |
| --- | --- |
| Echocardiographic left ventricular mass index (g/m^2^) | 116.2±47.3 |
| Echocardiogram Ejection Fraction (EF) (%) | 60.2±15.1 |
| Blood pressure (mmHg) Systolic/Diastolic | 162.5±29.6/92.7±15.9 |
| Pulse pressure (mmHg) | 71.2±20.6 |
| ABI^a^ | 0.9±0.1 |
| Brachial-ankle PWV^b^ (m/s) | 17.8±3.6 |
| Carotid-femoral PWV (m/s) | 12.5±3.0 |
| eGFR (ml/min/1.73m^2^) | 72.6±31.2 |
| Urinary ACR^c^ (µg/mg) | 138.2±147.5 |

**Notes:** ^a^ABI:ankle-Brachial Index^b^PWV: Pulse Wave Velocity^c^ ACR: Albumin-to-Creatinine Ratio

**Table S2.**Association of MMP2 gene polymorphism rs243865 (−1306C>T) and Carotid artery stenosis in resistant hypertension (n=78).

|  |  | **Carotid artery stenosis** | | **Univariate logistic regression** | | **Multivariate logistic regression^a^** | |
| --- | --- | --- | --- | --- | --- | --- | --- |
|  |  | Yes | No | OR^b^ (95% CI) | P value | OR(95% CI) | P value |
| Rs243865(−1306C>T) | T Carrier | 13(72.2) | 5(27.8) | 3.0 (1.0‐9.4) | .057 | 4.5 (1.1‐20.1) | .048 |
|  | CC | 28(46.7) | 32(53.3) | - |  | - |  |
| Age group | ≤60 years | 7 (25.9) | 20(74.1) | - |  | - |  |
|  | ≥61 years | 34(66.7) | 17(33.3) | 5.7 (2.1‐16.1) | .001 | 3.7 (1.1‐12.1) | .03 |
| Duration of | ≤10 years | 21(39.6) | 32(60.4) | - |  | - |  |
| Hypertension | ≥11 years | 20(80.0) | 5(20.0) | 6.1 (2.0‐18.7) | .002 | 3.6 (1.0‐12.7) | .050 |
| Sodium serum concentration | Mean ± SD)^c^ | 139±3.6 | 136±3.0 | 1.3 (1.1‐1.5) | .001 | 1.3 (1.1‐1.6) | .005 |

**Notes:** ^a^ The four-factor model R^2^=0.2857^b^OR: Odd ratio^c^SD: Standard deviation

**Table S3.** Association of MMP2 gene polymorphism rs243865 (-1306C>T) with Carotid-Femoral PWV in Resistant Hypertension (n=78).

|  |  | **n** | **Mean ± SD^a^** | **Univariate linear regression** | | **Multivariate linear regression^b^** | |
| --- | --- | --- | --- | --- | --- | --- | --- |
|  |  |  |  | **ß reg. coef. (95% CI)** | **p-value** | **ß reg. coef. (95% CI)** | **P value** |
| rs243865(-1306C>T) | T Carrier | 18 | 13.6±2.9 | 1.4 | .074 | 1.8 | .008 |
|  | CC | 60 | 12.1±2.9 | (-0.1‐3.0) |  | (0.5‐3.2) |  |
| Sex | Male | 29 | 11.6±2.8 | -1.42 | .04 | -1.1 | .074 |
|  | Femal | 49 | 13.0±2.9 | (-2.7)-(-0.06) |  | (-2.2)‐3.2 |  |
| Age group | ≤60 yrs | 27 | 10.9±2.9 | - |  | - |  |
|  | ≥61 yrs | 51 | 13.3±2.7 | 2.3 (1.01‐3.6) | .001 | 1.5 (0.3‐2.7) | .021 |
| Duration of  Hypertension | ≤10 years | 53 | 12.1±2.8 | - |  | 0.8 |  |
|  | ≥11 years | 25 | 13.5±3.1 | 1.4 (0.04‐2.8) | .049 | (-0.5)‐2.0 | .213 |
| Hypertension | Grade 1&2 | 53 | 11.6±2.6 | - |  |  |  |
| Level | Grade 3 | 25 | 14.3±2.8 | 2.7 (1.3‐3.9) | .001 | 2.7 (1.6‐3.9) | .001 |
| Diabetes | Yes | 22 | 13.4±3.1 | 1.3 | .08 | 0.3 |  |
|  | No | 56 | 12.1±2.8 | (-0.2)‐2.7 |  | (-0.9)‐1.5 | .657 |

**Notes:** ^a^SD: Standard deviation^b^ The six-factor model R^2^=0.3507
